# Influence of Scale Effect on Strength and Deformation Characteristics of Rockfill Materials

**DOI:** 10.3390/ma15155467

**Published:** 2022-08-08

**Authors:** Hongxing Han, Jing Li, Jicun Shi, Cuina Yang

**Affiliations:** 1 School of Civil Engineering and Architecture, Xinxiang University, Xinxiang 453003, China; 2 Architectural Teaching and Research Group, Xinxiang University of Broadcast & Television, Xinxiang 453000, China

**Keywords:** rockfill materials, scale effect, triaxial test, maximum particle size, confining pressure, deformation characteristics

## Abstract

The hybrid method was adopted to model the original gradation of rockfill materials. According to the specification requirements, three simulated gradations of rockfill materials have been obtained. By the same token, the corresponding maximum particle sizes are 20 mm, 40 mm and 60 mm, respectively. With samples prepared under the same criterion of relative density, the scale effect on strength and deformation characteristics of the rockfill materials were studied by large-scale and consolidated-drained triaxial compression tests. The results show that when the confining pressure is higher, the peak deviator stress decreases with the increase of the maximum particle size. With the increase of the maximum particle size, the cohesion of rockfill materials gradually increases and the internal friction angle gradually decreases. Under the condition of the same maximum particle size, with the increase of confining pressure, the volume strain at the phase transition increases gradually, while the stress ratio at the phase transition decreases. Under the same confining pressure, the larger the particle size is, the smaller the volume strain becomes and the lower the stress ratio at the phase transition is. Therefore, the research results can provide a theoretical basis for establishing the constitutive model of rockfill materials considering the scale effect.

## 1. Introduction

As the main filling material of earth rock dams, rockfill materials are widely used because they can be adapt to more complex geological conditions [1,2,3]. Block stone, crushed stone, stone slag, sandy soil, stone chips, backfill, and mixed soil containing a large number of coarse particles in cohesive soil are materials often used in engineering [4,5,6,7,8,9,10]. At present, with the continuous increase in the height of earth rock dams, the particle size of rockfill materials is also larger, some of which reach 600~800 mm, and some even reach 1000 mm [10,11]. Under the indoor triaxial test condition [2], the maximum particle size of the sample shall not exceed 1/5 of the sample diameter D. Limited by the size of the test instrument. The rockfill materials that exceed the maximum allowable particle size for the test must be reduced. There are great differences in the macro mechanical properties of rockfill materials replaced by scales of different sizes [3,10,11,12,13], which is the scale effect. In recent years, the research on scaling effect mainly focuses on two aspects [2]: on the one hand, it is to increase the sample size and restore the in situ size of rockfill as much as possible to reduce the impact of scaling [1,12,14]. On the other hand, it is to carry out research on the law of scaling effect, hoping to use the results of large- and medium-sized tests to deduce the mechanical properties of the original grade rockfill materials. Results of triaxial tests on rockfill materials by Rahmani and Panah [15] revealed that particle breakage increased with the increase maximum particle sizes of soft rockfill materials, and proposed a calculation method of relative crushing rate [2]. Another important factor for rockfill materials breakage is the critical state behavior of constituting rockfill aggregates [16]. In these studies, the fractal dimension proposed by Han [17] was used to evaluate the relationship between particle distribution and its mechanical properties. However, the maximum particle size of the sample is closely related to its mechanical properties [18]. The distribution of the particle size of rockfill has a great impact on its strength and deformation characteristics. Yang et al. [19] used a large triaxial apparatus to conduct triaxial tests on rockfill. The study found that the volume strain of the aggregate decreased with the increase of the maximum particle size, and the peak deviator stress increased with the increase of the maximum particle size. The parallel gradation and combination methods [20] are widely acknowledged in testing and designing of rockfill, inevitably instigating the use of reduced maximum particle size. Ventini et al. [21] studied the influence of gradation on the deformation characteristics of rockfill and believed that the volumetric strain of the two rockfill materials under low stress was similar to that of saturated soil samples. The research shows that the mechanical properties of the reduced alternative material are quite different from those of the original granular material [15,16,17,18,19,20,21], but the current understanding is not enough to quantitatively evaluate its impact, so it is necessary to conduct in-depth discussion.

In view of this, this paper has been used the hybrid method to reduce the original gradation into three simulated gradations according to the specification, and the corresponding maximum particle sizes are 20 mm, 40 mm and 60 mm, respectively. The same relative density is used as the sample preparation standard, and the ylsz30-3 large-scale dynamic and static triaxial apparatus is used to conduct isobaric consolidation drainage shear tests under different confining pressures on three simulated gradations. The test results are analyzed to study the influence of scaling effect on the strength and deformation characteristics of rockfill.

## 2. Experimental Details

### 2.1. Experimental Equipment

**Figure 1:**
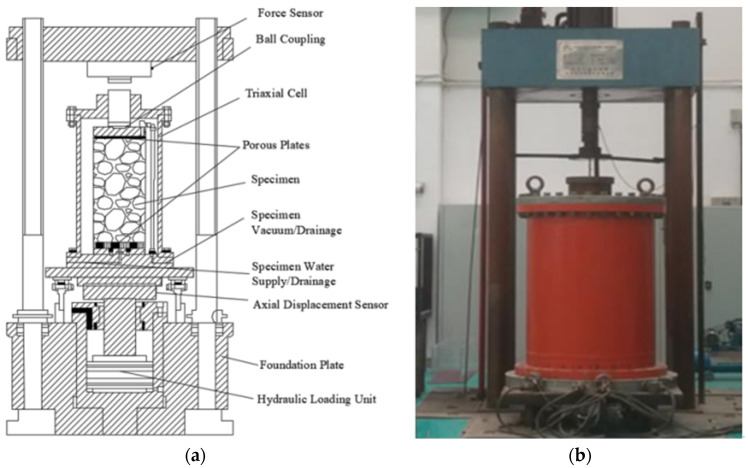
Large-scale triaxial compression apparatus: (**a**) overall view of the apparatus; and (**b**) pressure stabilization techniques.

A YLSZ30-3 large dynamic and static triaxial testing machine was used in this test. Sample size is ϕ300 mm ×600 mm, as shown in Figure 1. The equipment is used to study the strength and deformation characteristics of dam materials under different stress paths. The main technical parameters are: maximum axial load 2500 kN, maximum axial vertical deformation 300 mm, maximum confining pressure 6.0 MPa, and shear speed 0.2–2 mm/min. Strain control is adopted in the test, and the ratio of the maximum allowable particle size of the sample to the diameter of the sample is $d_{\max}/D=0.2$.

### 2.2. Experimental Materials

**Figure 2:**
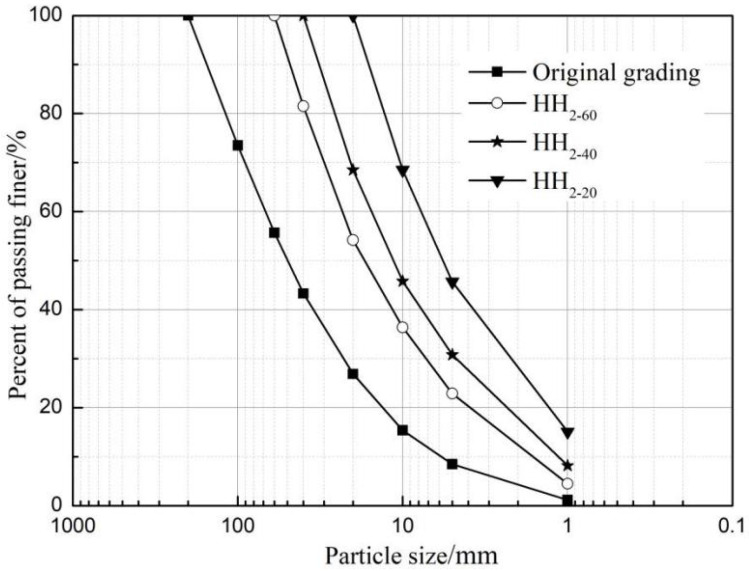
Grain-size distribution curves of rockfill materials.

The rockfill materials studied in this paper were selected from the main rockfill material of a rockfill dam. The lithology was weathered granite and the rock block was hard. The maximum particle size is 200 mm, the particle proportion is 2.76, the nonuniformity coefficient is $C_{u}=11.68$, and the curvature coefficient is $C_{c}=1.31$. Using mixed method to reduce the prototype grading of rockfill. The number is HH. The maximum particle size of the simulated gradation is 60, 40 and 20 mm, respectively, which is used as the subscript of the number to distinguish. For example, HH_2-60_ represents the reduction multiple n=2, and the maximum particle size after the reduction is 60 mm. Figure 2 shows grain-size distribution curves of rockfill materials. The simulated grading and results of rockfill materials are shown in Table 1.

**Figure 3:**
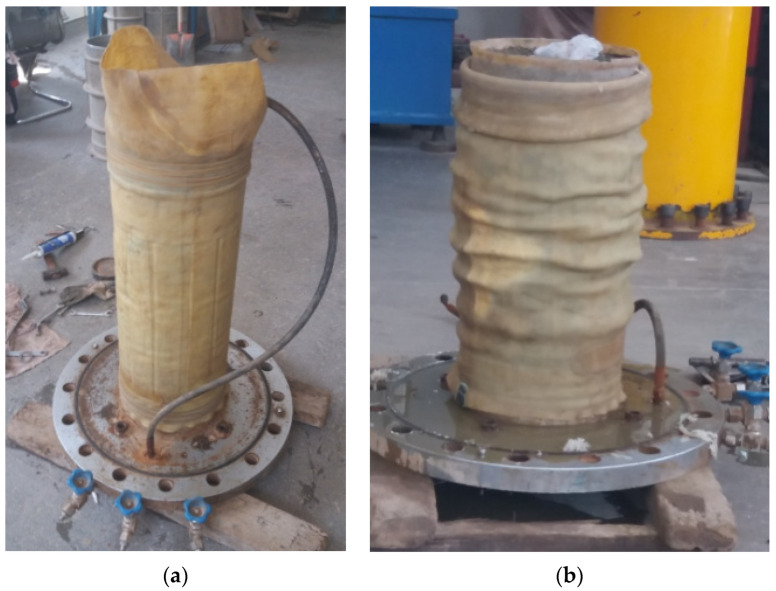
Typical photographs of the sample before and after the test ($\sigma_{3}=600$ kPa). (**a**) Loading sample. (**b**) Failure specimen.

The sample is prepared by layered vibration method, 3 layers, each layer of 20 cm, compacted to the design height by surface oscillator, and the frequency is set to 50 Hz. The prepared samples are saturated by filling water at the bottom and pumping air at the top. After consolidation, they are sheared at a strain rate of 1 mm/min. There are three groups of confining pressure tests. The test control conditions are shown in Table 2. The test termination condition is that the axial strain of the specimen is 15%. Typical photographs of the sample before and after the test are shown in Figure 3.

## 3. Results and Discussion

### 3.1. Strength Characteristic Analysis

**Figure 4:**
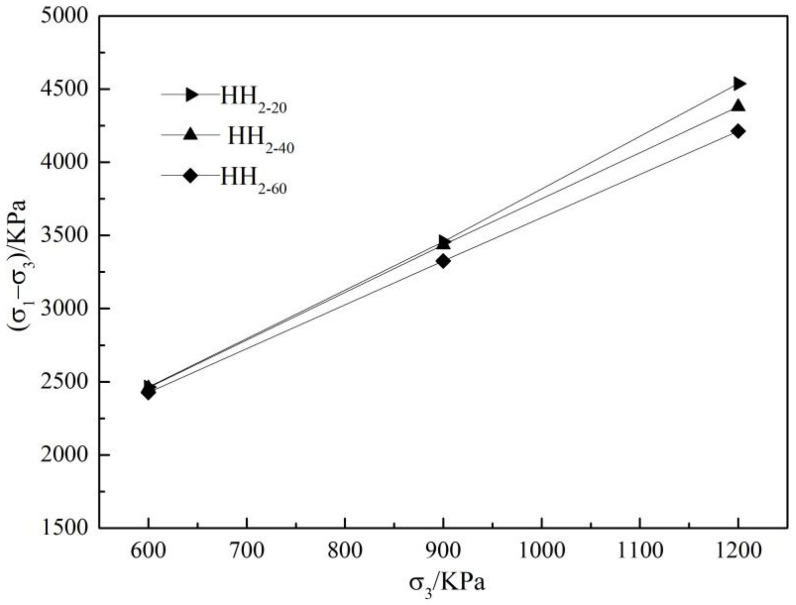
The relationship between peak strength and confining pressure.

Three groups of rockfill materials are obtained by the hybrid scale method. The relationship curve between peak deviator stress ${\left(\sigma_{1}-\sigma_{3}\right)}_{f}$ and confining pressure $\sigma_{3}$ of each group samples under different confining pressures is shown in Figure 4. It can be seen from Figure 4 that under the same confining pressure, the peak deviator stress of HH_2-20_ material is the largest and that of HH_2-60_ material is the smallest; under the same maximum particle size, the peak intensity increases with the improvement of confining pressure, which is approximately linear.

Rockfill materials are cohesionless soil, but gravel, pebble and rockfill are closely meshed with each other under dense conditions, and there is a “biting force”, which is similar to the cohesion of cohesive soil in macro. Table 3 lists the strength index of different size values. It can be seen from Table 3 that the cohesion increases with the increase of the maximum particle size of rockfill materials, while the internal friction angle decreases with the increase of the maximum particle size.

The edges and corners of particles with larger particle size are relatively sharp. The more prone it is to stress concentration, the more likely the particles are to be broken. After failure, the particle size stress is redistributed, and the particle size connection stress becomes weak, so the internal friction angle of rockfill materials increases.

### 3.2. Analysis of Deformation Characteristics

**Figure 5:**
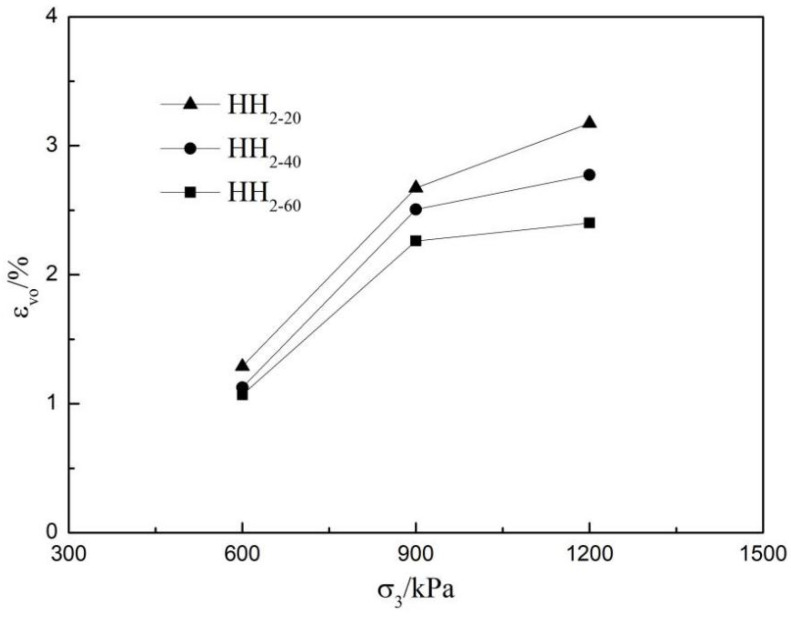
Relationship between volume strain $\varepsilon_{v0}$ and confining pressure $\sigma_{3}$ at phase change.

The content of coarse material is more after that the coarse material is reduced by mixing method and induced large shrinkage deformation [22]. In order to further study the influence of the maximum particle size $d_{\max}$ of rockfill materials on its dilatancy, this paper supplies the relationship between the volume strain value $\varepsilon_{v0}$ and the confining pressure $\sigma_{3}$ of the rockfill specimen at the phase change position (the turning point of volume from compression to expansion) under different confining pressures of three groups after scale reduction, as shown in Figure 5. It can be seen from Figure 5 that the volume strain values $\varepsilon_{v0}$ at the phase transformation of three groups of samples increase with the increase of confining pressure $\sigma_{3}$. Under the same confining pressure, the volume strain $\varepsilon_{v0}$ obtained by HH_2-60_ scale method is at the bottom side, however that obtained by HH_2-20_ scale method is at the top side, and that obtained by HH_2-40_ scale method is between the HH_2-20_ and HH_2-60_. The larger the maximum particle size $d_{\max}$ is, the larger the volume strain $\varepsilon_{v0}$ is.

**Figure 6:**
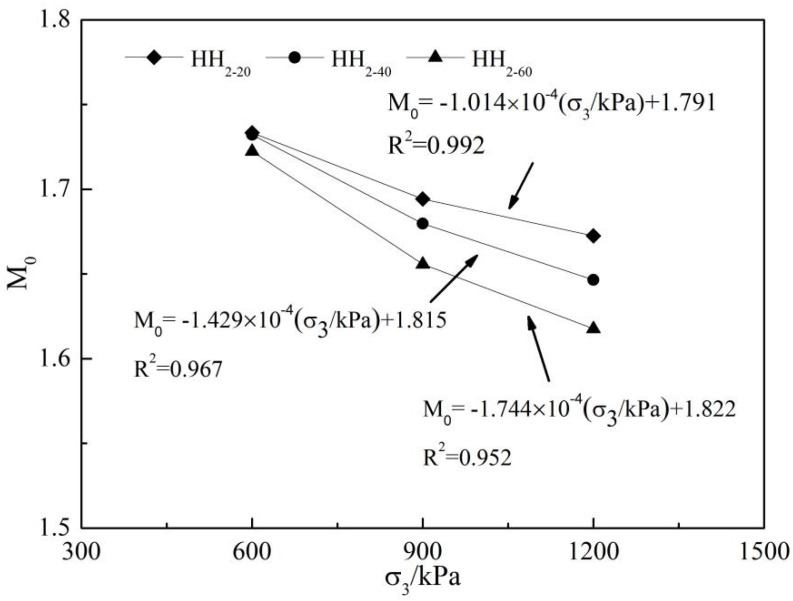
Relationship between stress ratio $M_{0}$ and confining pressure $\sigma_{3}$ at phase change.

The accurate description of dilatancy is very important for the constitutive model of dilatancy. When some shear expansion models are established, the stress ratio at the transformation ($M_{0}$, $M_{0}=q_{0}/{p^{\prime }}_{0}$, where $q_{0}$ is the generalized shear stress at the transformation, ${p^{\prime }}_{0}$ is the average principal stress at the transformation) is often used to express the shear expansion equation or hardening parameters. Three groups of test data are sorted out, and the relationship between $M_{0}$ and $\sigma_{3}/\mathrm{kPa}$ is drawn, as shown in Figure 6. From Figure 6, it can be seen that the stress ratio $M_{0}$ of rockfill samples at the transformation point decreases with the increase of confining pressure $\sigma_{3}$ after three groups of downscales, and a good linear relationship is formed with the confining pressure. The following formula can be used for fitting:

(1) $$M_{0}=a\times \frac{\sigma_{3}}{\mathrm{kPa}}+b$$

where: a, b are test parameters, see Figure 6 for specific values.

### 3.3. Nonlinearity of Shear Strength

The shear strength of soil is the resistance of soil to the shear stress produced by external load. In 1776, based on a large number of experiments, the famous Coulomb formula was proposed [23].

(2) $$\tau_{f}=c+\sigma_{n}\tan\varphi$$

where: $\tau_{f}$ is shear strength of damaged surface, $\sigma_{n}$ is normal stress of damaged surface; c is cohesive force, φ is internal friction angle.

Duncan has developed the hyperbolic stress–strain model on the basis of Coulomb [24], and adopted the logarithmic form for the strength envelope of cohesionless soil bending, the expression is as follows.

(3) $$\varphi =\varphi_{0}-\Delta \varphi \mathrm{lg}\left(\frac{\sigma_{3}}{P_{a}}\right)$$

where: $\sigma_{3}$ is the small main stress, $p_{a}$ is the atmospheric pressure, $\varphi_{0},\;\Delta \varphi$ is the material parameter.

**Figure 7:**
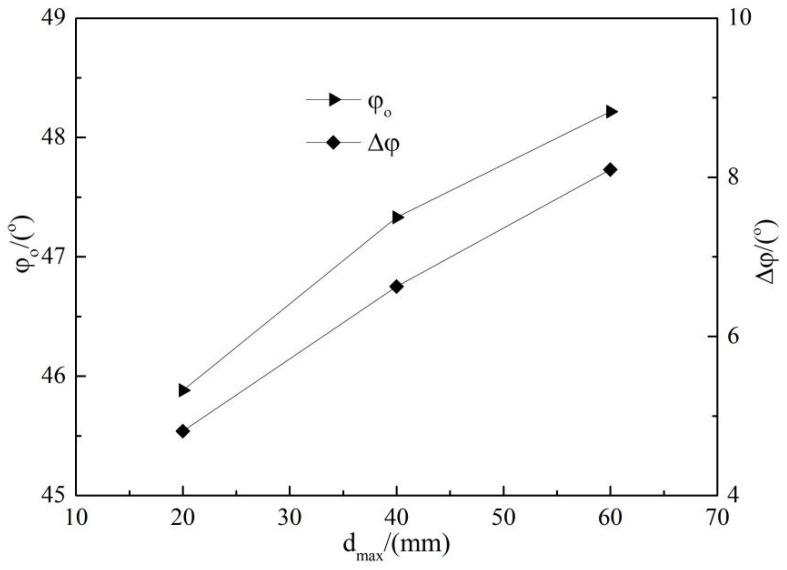
Relationship between $\varphi_{0},\;\Delta \varphi$ and $d_{\max}$.

Figure 7 shows the relationship between nonlinear strength index $\varphi_{0}$, Δφ and $d_{\max}$. It can be seen from Figure 7 that the nonlinear strength index $\varphi_{0}$ and Δφ both increases with the increase of the maximum particle size $d_{\max}$. Among them, the value $\varphi_{0}$ and Δφ of HH_2-40_ increases by 1.449° and 1.818° compared with the corresponding value of HH_2-20_, while the value of HH_2-60_ increases by 0.885° and 1.470° compared with the corresponding value of HH_2-40_. When the particles are larger, the biting force between soil particles is better, and the correlation with $d_{\max}$ is weaker.

## 4. Conclusions

(1) Under the same confining pressure, the smaller the maximum particle size $d_{\max}$ is in the three groups of granular materials, the larger the corresponding peak deviator stress ${\left(\sigma_{1}-\sigma_{3}\right)}_{f}$, the volume strain $\varepsilon_{vo}$ and stress ratio $M_{0}$ are at the phase transformation, which indicates that the soil particles have a higher overturning capacity, and the macroscopic performance is that HH_2-20_ material has a stronger dilatancy.
(2) With the increase of confining pressure $\sigma_{3}$, the peak deviator stress ${\left(\sigma_{1}-\sigma_{3}\right)}_{f}$ and volume strain $\varepsilon_{vo}$ in the three groups of granular materials gradually increased, while the stress ratio $M_{0}$ at the phase transformation gradually decreased, and the stress ratio $M_{0}$ at the phase transformation showed a good linear relationship with the confining pressure $\sigma_{3}$.
(3) In the linear shear strength index, with the increase of the maximum particle size $d_{\max}$, the cohesion of rockfill materials *c* gradually increases, while the internal friction angle φ of rockfill materials shows a downward trend. Among the nonlinear shear strength indexes, the strength indexes $\varphi_{0}$ and Δφ in the three groups of granular materials increase with the increase of the maximum particle size $d_{\max}$.

## Figures and Tables

**Table 1:** The test gradation of rockfill materials and tested results.

Hybrid Method	Effective Particle Size d_10_	Intermediate Particle Size d_30_	Controlled Particle Size d_60_	Nonuniformity Coefficient *C*_u_	Curvature Coefficient *C*_c_
HH_2-60_	2.89	9.86	24.55	8.49	1.37
HH_2-40_	2.89	8.41	18.68	6.46	1.31
HH_2-20_	2.89	6.69	11.47	3.97	1.35

**Table 2:** The test plan.

Hybrid Method	Sample Diameter/mm	$d_{\max}/(\mathrm{mm})$	$\rho_{d}/(G\cdot {\mathrm{cm}}^{-3})$	$\sigma_{3}/(\mathrm{KPa})$
HH_2-60_	300	60	2.181	600, 900, 1200
HH_2-40_	300	40	2.155	600, 900, 1200
HH_2-20_	300	20	2.114	600, 900, 1200

**Table 3:** Strength index of different size value.

Hybrid Method	$\sigma_{3}/\mathrm{kPa}$	${\left(\sigma_{1}-\sigma_{3}\right)}_{f}/\mathrm{kPa}$	c/kPa	φ $/({}^{ο})$
HH_2-20_	600	2463.2	125.37	38.33
	900	3456.3		
	1200	4535.8		
HH_2-40_	600	2459.4	148.01	37.69
	900	3435.1		
	1200	4379.2		
HH_2-60_	600	2426.3	166.87	36.74
	900	3325.5		
	1200	4212.7		

## Data Availability

All data used to support the findings of this study are available from the corresponding author upon request.
